# Impact of Case Definitions on Efficacy Estimation in Clinical Trials—A Proof-of-Principle Based on Historical Examples

**DOI:** 10.3390/antibiotics9070379

**Published:** 2020-07-04

**Authors:** Andreas Hahn, Hagen Frickmann, Andreas E. Zautner

**Affiliations:** 1 Institute for Medical Microbiology, Virology and Hygiene, University Medicine Rostock, 18057 Rostock, Germany; hahn.andreas@me.com; 2 Department of Microbiology and Hospital Hygiene, Bundeswehr Hospital Hamburg, 20359 Hamburg, Germany; 3 Institut für Medizinische Mikrobiologie, Universitätsmedizin Göttingen, 37075 Göttingen, Germany; azautne@gwdg.de

**Keywords:** efficacy estimation, case definition, specificity, clinical trial

## Abstract

Efficacy estimations in clinical trials are based on case definitions. Commonly, they are a more or less complex set of conditions that have to be fulfilled in order to define a clinical case. In the simplest variant, such a case is identical with a single positive diagnostic test result. Frequently, however, case definitions are more complex. Further, their conditions often ignore the inherent logical structure of symptoms and disease: A symptom or a set of symptoms may be necessary but not sufficient for the unambiguous identification of a case. After describing the structure of case definitions and its impact on efficacy estimations, we exemplify this impact using data from two clinical trials dealing with the effectiveness of the vaginal application of tenofovir gel for the prevention of HIV infections and with the therapeutic effects of fecal transplantation on recurrent *Clostridium difficile* infections. We demonstrate that the diagnostic performance of case definitions affects efficacy estimations for interventions in clinical trials. The potential risk of bias and uncertainty is high, irrespective of the complexity of the case definition. Accordingly, case definitions in clinical trials should focus on specificity in order to avoid the risk of bias.

## 1. Introduction

The endpoints of clinical trials are usually defined by case definitions. Recently, the effect of diagnostic test specificity on the reliability of study outcomes has been demonstrated [1]. In this paper, we show that it is not only test specificity but the combined specificity of both diagnostic tests and clinical case definitions that defines the impact on the study results, making proper handling of both elements, with focus on specificity, advisable prior to conducting a clinical trial.

To demonstrate this hypothesis, we have chosen two well-known historical studies on the specific effects of antimicrobial interventions. The first, the CAPRISA 004 trial, described a double-blind randomized controlled trial on the preventive effects of vaginal tenofovir gel against HIV infection, showing only a moderate effect [2]. With HIV infection as the study endpoint, the case definition is identical with the diagnostic test results, describing the simplest situation. In contrast, the widely referenced study by van Nood and colleagues on the effects of fecal transplantation for the treatment of recurrent *Clostridium difficile* infections [3] was chosen as an example with a slightly more complex and less-specific endpoint. This is because the endpoint, *C. difficile*-associated gastroenteritis, depends on both the diagnostic detection of the pathogen (i.e., the test component) and the causal attribution of the pathogen to clinical disease (i.e., the case definition component). The latter point is not trivial because, as recently reviewed, determining the etiological relevance of a pathogen in patients with diarrhea may indeed be challenging [4].

Based on these two examples, the effects of the specificity of case definitions are analyzed in a modeling approach.

## 2. Materials and Methods

If one were to look at the set of all an individual’s possible symptoms or attributes, then its power set would be the set of all possible combinations of these symptoms or attributes. A case definition in a very general sense is then any of these combinations:

Let Ω be the set of all symptoms and attributes and P(Ω) its power set. Then, every subset C⊆P(Ω) is called a case definition C

If there is a case definition (e.g., for a disease) and a set of individuals, then a case is an individual whose symptoms and attributes fulfill the case definition:

Let I be the set of all individuals. $S_{k}\in P\left(\Omega \right)$ denotes the individual set of symptoms and attributes for individual $i_{k}\in I$. A case is defined by Equation (1):

Case: I×P(Ω)→{0,1}

with

(1) $$Case\left(i_{k},\;S_{k}\right):=\{\begin{array}{c} 1\;if\;\exists c\in C:c\subseteq S_{k}\\ 0\;else \end{array}$$

Commonly, the symptoms and attributes of a case definition C⊆P(Ω) are the result of a disease. Then

Disease⇒C⊆P(Ω)⇔Disease⊆C⊆P(Ω)

holds.

This means that if the symptoms and attributes of a case definition are a result of the disease of interest, then the set of diseased individuals is always a subset of the set of individuals fulfilling the case definition.

Generally, this is not equivalent to the disease itself. In particular,

C⊆P(Ω)⇒Disease⇔C⊆P(Ω)⊆Disease

does not hold generally. Therefore, a case is not necessarily diseased. Thus, the true disease status is unknown, even in case of a positive case definition.

The true but unknown disease status for every individual $i_{k}\in I$ is given by Equation (2):

Disease status: I→{0,1}

with

(2) $$Disease\;status\left(i_{k}\right):=\;\{\begin{array}{c} 1\;if\;i_{k}\;diseased\\ 0\;if\;i_{k}\;not\;diseased \end{array}$$

Since the set of individuals fulfilling a case definition of a specific disease and the set of specifically diseased individuals are in general not the same, a case definition can be understood as a diagnostic test for its disease.

Then, the sensitivity of a case definition is given by Equation (3):

Se:I→[0,1]

with

(3) $$\frac{\left|\left\{i_{k}:Disease\;status\;\left(i_{k}\right)=1\right\}\cap \left\{i_{k}:\;Case\;\left(i_{k}\right)=1\right\}\right|}{\left|\left\{i_{k}:Disease\;status\;\left(i_{k}\right)=1\right\}\right|}$$

In the same way, the specificity of a case definition is given by Equation (4):

Sp:I→[0,1]

with

(4) $$\frac{\left|\left\{i_{k}:Disease\;status\;\left(i_{k}\right)=0\right\}\cap \left\{i_{k}:\;Case\;\left(i_{k}\right)=0\right\}\right|}{\left|\left\{i_{k}:Disease\;status\;\left(i_{k}\right)=0\right\}\right|}$$

Obviously, the definitions above lead to the known definitions for sensitivity and specificity:

Se (Case definition)=P(Case=1|Disease status=1)

and

Sp (Case definition)=P(Case=0|Disease status=0)

A case definition C is perfectly sensitive if the set of diseased individuals is a subset of all cases:

$$\left\{i_{k}:Disease\;status\;\left(i_{k}\right)=1\right\}\subseteq \left\{i_{k}:Case\left(i_{k}\right)=1\right\}$$

This is the same as:

Disease⇒C⊆P(Ω)

A case definition C is completely specific if the set of non-diseased individuals is a subset of all non-cases:

$$\left\{i_{k}:Disease\;status\;\left(i_{k}\right)=0\right\}\subseteq \left\{i_{k}:Case\left(i_{k}\right)=0\right\}$$

If a case definition is completely specific, then we have:

C⊆P(Ω)⇒Disease

Only if a case definition is completely sensitive as well as completely specific is it equivalent to the disease:

C⊆P(Ω)⇔Disease

This means that the set of diseased individuals is identical with the set of individuals fulfilling the case definition.

Since every set of symptoms or attributes should be verified by a diagnostic test system that has its own sensitivity and specificity as a combination of all individual sensitivities and specificities of every component of the diagnostic test system, our initial definition of a case has to be modified as follows:

Let I be the set of all individuals. $S_{k}\in P\left(\Omega \right)$ denotes the individual set of symptoms and attributes for individual $i_{k}\in I$. Let the diagnostic test system be a map from the power set of symptoms and attributes to the tuple (0,1), where 0 indicates a negative and 1 indicates a positive diagnostic test result:

Diagnostic test system: P(Ω)→{0,1}

A case is defined by Equation (5):

Case: I×P(Ω)→{0,1}

with

(5) $$Case\left(i_{k},\;S_{k}\right):=\;\{\begin{array}{c} 1\;if\;\exists c\in C:c\;\subseteq \;S_{k}\;with\;Diagnostic\;test\;system\left(c\right)=1\\ 0\;else \end{array}$$

This means that a case is an individual for whom there exists a set of symptoms or attributes of the case definition for whom the diagnostic test system conducted will be positive.

The impacts of diagnostic sensitivity and specificity on prevalence or intervention efficacy estimations were described by Gart and Buck [5,6] and Rogan and Gladen [7] as well as Lachenbruch [8], Gart [9], and Neyman [10]. They proposed sensitivity- and specificity-adjusted point estimators for prevalence or incidence and intervention efficacy. While the authors above focused specifically on diagnostic tests, we now point out that even a case definition itself has its own sensitivity and specificity regarding the disease for which the case definition has been defined. Consequently, the overall sensitivity and specificity of a case definition is always a combination of the sensitivity and specificity of the test system testing for the attributes and symptoms of the case definition, as well as the sensitivity and specificity of the case definition itself.

If overall sensitivity and specificity are known, then it is possible to apply the sensitivity- and specificity-adjusted point estimators of the authors quoted above.

Given a case definition C of a disease with sensitivity Se and specificity Sp, then the sensitivity- and specificity-adjusted prevalence estimator Prev is given by Equation (6):

(6) $$Prev=\frac{\frac{\sum_{k=1}^{n}Case\left(i_{k},\;S_{k}\right)}{n}-1+Sp}{Se+Sp-1}$$

Based on the estimator above in (6), a sensitivity- and specificity-adjusted prevention efficacy PE estimator is given by Equation (7):

(7) $$PE=1-\frac{\frac{\sum_{k=1}^{n}Case\left(i_{k},\;S_{k}\right)}{n}-1+Sp}{\frac{\sum_{l=1}^{n}Case\left(i_{l},\;S_{l}\right)}{n}-1+Sp}$$

The estimator above in (7) holds for the specificity of a case definition that is constant over the study arms. Obviously, specificity of the case definition is fundamental for the bias in an unadjusted estimation that uses the raw rate of cases in both arms for efficacy estimation. This explains why case definitions in clinical trials may differ from case definitions in the clinical context. Clinical trials are designed to estimate unbiased effects in a population, while the focus of a clinical case definition is the individual patient.

Case definitions should be designed to be specific, considering their focus in clinical trials. Furthermore, variation of specificity over the study arms should be avoided. Accordingly, an open-label design in accordance with investigator-assessed endpoints or patient-reported outcomes should be avoided.

For cases where the sensitivity or specificity of a case definition vary over study arms, an adjusted estimator is given by Equation (8):

(8) $$\frac{\left(\frac{\sum_{k=1}^{n}Case\left(i_{1k},\;S_{1k}\right)}{n}-1+Sp_{1}\right)\left(Se_{2}+Sp_{2}-1\right)}{\left(\frac{\sum_{l=1}^{n}Case\left(i_{2l},\;S_{2l}\right)}{n}-1+Sp\right)\left(Se_{1}+Sp_{1}-1\right)}$$

The 0.95 confidence intervals are given by Equation (9) as follows:

$$0.95\;CI=1-e^{Ln\left(1-IE\right)\pm 1.96\left(\frac{1}{prev_{1}^{2}}Var\left(Prev_{1}\right)+\frac{1}{prev_{2}^{2}}Var\left(Prev_{2}\right)\right)}$$

with

(9) $$Var\left(Prev\right)=\frac{\frac{\sum_{k=1}^{n}Case\left(i_{k},\;S_{k}\right)}{n}\left(1-\frac{\sum_{k=1}^{n}Case\left(i_{k},\;S_{k}\right)}{n}\right)}{n\;{\left(Se+Sp-1\right)}^{2}}$$

One of the main obstacles for applying the sensitivity- and specificity-adjusted estimators above is that the diagnostic sensitivity and specificity have to be known. Since the true disease status is likely unknown, indirect methods are often used for the evaluation of diagnostic sensitivity and specificity. Methods and problems regarding the assumptions of such approaches have been widely discussed [10,11,12,13,14,15,16,17,18,19,20].

The validity of indirect methods for the estimation of the diagnostic sensitivity and specificity thus depends on the fulfillment of the underlying model assumptions. Ideally, the case definition should be defined with a specificity virtually equal to 1, and it should not vary over treatment arms, leading to a minimum of bias as a consequence of Equation (7).

### 2.1. Summaries of Historical Studies Used to Exemplify the Approach

#### 2.1.1. The CAPRISA 004 Trial

The case definitions of the CAPRISA 004 trial on the preventive effects of vaginal tenofovir gel against HIV infection [2] and, in comparison, the study by van Nood and colleagues on the effects of fecal transplantation for the treatment of recurrent *Clostridium difficile* infections [3] are analyzed and discussed.

The double-blind, randomized, controlled CAPRISA 004 trial assessed effectiveness and safety of a 1% vaginal gel formulation of tenofovir for preventing the acquisition of HIV. It was conducted to compare tenofovir gel (*n* = 445) with placebo gel (*n* = 444) in sexually active, non-HIV-infected 18–40-year-old women in urban and rural KwaZulu-Natal, South Africa. At monthly follow-up visits for 30 months, the parameters HIV serostatus, safety, sexual behavior, and gel and condom use were assessed. In the tenofovir gel arm, reported HIV incidence was 5.6 per 100 woman-years (wy, 38/680.6 wy), compared to 9.1 per 100 wy (60/660.7 wy) in the placebo arm. The overall protective efficacy against HIV infection was estimated at 39%. Two HIV rapid tests, Determine^®^ HIV-1/2 (Abbott Laboratories, IL, USA) and Uni-Gold Recombigen^®^ HIV test (Trinity Biotech, Wicklow, Ireland), were applied during each study visit. By protocol, only HIV infections during study follow-up in eligibly enrolled women, as confirmed by two independent RNA PCR results, were defined as study endpoints. Participants in the HIV window period at the end of the study were included as HIV-related endpoints if seropositivity was confirmed after the study.

#### 2.1.2. The Study by van Nood and Colleagues

The study by van Nood and colleagues investigated the effect of duodenal infusion of donor feces in patients with recurrent *C. difficile* infection. The study patients were randomly assigned to receive one of three therapeutic approaches:

- An initial vancomycin regimen of 500 mg orally four times per day for 4 days, followed by bowel lavage and subsequent infusion of a solution of donor feces through a nasoduodenal tube;
- A standard vancomycin regimen of 500 mg orally four times per day for 14 days; or
- A standard vancomycin regimen with bowel lavage.

The primary endpoint was the resolution of diarrhea associated with *C. difficile* infection without relapse after 10 weeks. For this purpose, “resolution of diarrhea associated with *C. difficile* infection” was defined as the absence of diarrhea or persistent diarrhea that could be explained by other causes with three consecutive negative stool tests for *C. difficile* toxin.

## 3. Results and Discussion

In the following we demonstrate the effect of case definition for the two historical clinical trials described above.

### 3.1. Effectiveness and Safety of Tenofovir Gel, an Antiretroviral Microbicide, for the Prevention of HIV Infection in Women

The structure of the case definition in this study is simple since there is only one attribute to be confirmed—An HIV infection after inclusion into the study. Thus, a case according to protocol (see also Equation (10)) was defined as a woman who was HIV-negative at the time of inclusion in the study with four positive test results indicating HIV infection (two positive HIV rapid tests and two positive RNA PCR results) in the course of the study:

Case: I×P(Ω)→{0,1}

with

(10) $$Case\left(i_{k},\;S_{k}\right):=\;\{\begin{array}{c} 1\;if\;all\;4\;conducted\;HIV\;tests\;of\;the\;individuum\;i_{k}\;are\;positive\\ 0\;else \end{array}$$

As such, the overall sensitivity and specificity are given by the diagnostic test sensitivity and specificity of the four diagnostic tests according to Equations (11) and (12):

(11) $$Sensitivity_{overall}=\overset{4}{\underset{i=1}{\prod }}Sensitivity_{i}$$

(12) $$Specificity_{overall}=1-\overset{4}{\underset{i=1}{\prod }}\left(1-Specificity_{i}\right)$$

For the Determine^®^ HIV-1/2 rapid test (Abbott Laboratories), sensitivity of 0.998 and specificity of 0.994, and for the Uni-Gold Recombigen^®^ HIV test (Trinity Biotech), sensitivity of 0.985 and specificity of 0.995 were reported by Piwowar-Manning and colleagues [21]. The diagnostic performance of the PCRs conducted is not reported. Nevertheless, it can be assumed that the sensitivity and specificity of these PCRs are very high. Assuming that the PCR specificity is 0.99 or higher, the overall specificity of the case definition is practically 1. Using Equation (7) from the Materials and Methods section, it follows that the case definition leads to a practically unbiased efficacy estimation even without any adjustment (see also Equation (13)):

(13) $$PE=1-\frac{\frac{\sum_{k=1}^{n}Case\left(i_{k},\;S_{k}\right)}{n}-1+Sp}{\frac{\sum_{l=1}^{n}Case\left(i_{l},\;S_{l}\right)}{n}-1+Sp}=1-\frac{\frac{\sum_{k=1}^{n}Case\left(i_{k},\;S_{k}\right)}{n}-1+1}{\frac{\sum_{l=1}^{n}Case\left(i_{l},\;S_{l}\right)}{n}-1+1}=1-\frac{\frac{\sum_{k=1}^{n}Case\left(i_{k},\;S_{k}\right)}{n}}{\frac{\sum_{l=1}^{n}Case\left(i_{l},\;S_{l}\right)}{n}}$$

**Figure 1:**
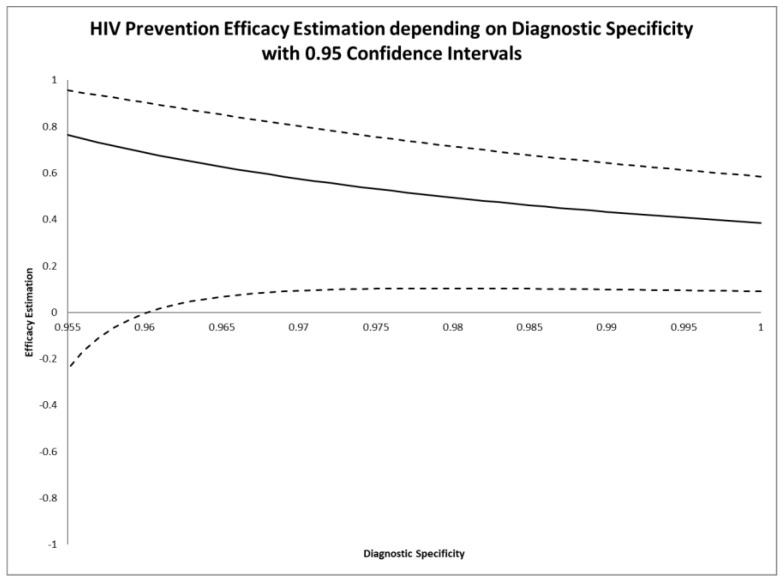
Adjusted efficacy estimates (*y*-axis) depending on endpoint specificity (*x*-axis) applying Equation (7) with 0.95 confidence intervals and using Equation (9) for the primary endpoint of the CAPRISA 004 trial.

Nevertheless, in Table 1 and Figure 1, we illustrate the variation of adjusted efficacy estimates with 0.95 confidence intervals calculated by Equation (9) for varying specificity assumptions. The unadjusted estimation is identical with the case of ideal diagnostic specificity that can be assumed for this trial. Therefore, unadjusted and adjusted estimations are the same if a diagnostic specificity of 1 can be assumed.

Although CAPRISA 004 was among the least-efficient HIV pre-exposure prophylaxis (PrEP) trials [22,23] for various reasons, with a major limitation in the field of adherence [24,25], the case definition was very well chosen, and later trials confirmed the preventive usefulness of the HIV PrEP approach with similar definitions of the clinical endpoint [26,27].

### 3.2. Duodenal Infusion of Donor Feces for Recurrent Clostridium difficile

The case definition based on the endpoint “resolution of diarrhea associated with *C. difficile* infection,” meaning an individual who was initially cured had a relapse within 10 weeks after initiation of therapy, can be reduced to “diarrhea” and “at least one positive test out of three tests for *C. difficile* toxin.” (see also Equation (14))

Case: I×P(Ω)→{0,1}

with

(14) $$Case\left(i_{k},\;S_{k}\right):=\;\{\begin{array}{c} 1\;if\;i_{k}\;has\;diarrhea\;and\;at\;least\;1\;of\;3\;conducted\;tests\;\\ for\;C.difficile\;is\;positive\\ 0\;else \end{array}$$

Assuming that the diagnosis of diarrhea as a clinical symptom is always true, the overall sensitivity and specificity of the case definition depend on the test system for *C. difficile* toxin. Its sensitivity and specificity (see also Equations (15) and (16)) are given by:

(15) $$Sensitivity_{overall}=1-\overset{3}{\underset{i=1}{\prod }}\left(1-Sensitivity_{i}\right)$$

(16) $$Specificity_{overall}=\overset{3}{\underset{i=1}{\prod }}Specificity_{i}$$

**Figure 2:**
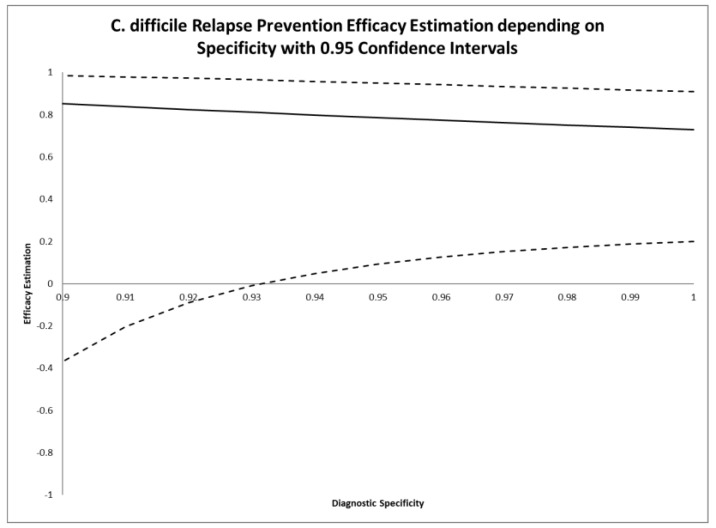
Adjusted efficacy estimates (*y*-axis) depending on endpoint specificity (*x*-axis) applying Equation (7) with 0.95 confidence intervals and using Equation (9) for the primary endpoint of the study by van Nood and colleagues on the effects of fecal transplantation for the treatment of recurrent *Clostridium difficile* infections.

The reported sensitivity and specificity for the test conducted (Premier Toxin A and B Assay) were 0.9744 and 0.9752 as reported by Novak-Weekley and colleagues [28]. This leads to an overall endpoint specificity of 0.927 and an overall endpoint sensitivity of 0.99998. Compared with the CAPRISA 004 trial, the case definition of this trial leads to a maximum endpoint sensitivity, while the endpoint specificity is reduced. As follows from Equation (7) from the Materials and Methods section, the reduced specificity leads to a bias in efficacy estimation. As presented in Table 2 and Figure 2, where adjusted efficacy estimations with 0.95 confidence intervals are given for varying endpoint specificity, the overall specificity of 0.927 leads to a loss of significance for the adjusted efficacy estimation. Contrary to the HIV trial modeled above, the case definition in this trial does not result in a maximum specificity that would lead to an unbiased efficacy estimation even without adjustment. A review of controversies associated with therapeutic fecal transplantation was published by van Nood and colleagues one year after the publication of the study addressed in this modeling [29]. The allocation of etiological relevance to pathogens in human samples remains an issue of ongoing debate [30,31,32].

As mentioned above, case definitions can be described as subsets of the power set of the set of all possible human attributes including clinical symptoms and laboratory parameters. Since clinical symptoms especially are a result of a disease and not the disease itself, the inference of a disease from a symptom is logically problematic. It follows that case definitions have their own sensitivity and specificity, just as classical diagnostic methods do [1]. From Lachenbruch’s sensitivity- and specificity-adjusted efficacy estimator [8], it also follows that the diagnostic specificity in particular is critical for the bias of an unadjusted estimation.

Since varying sensitivity and specificity over study arms makes the situation much more complex, the study design is also very critical. For example, open-label studies with subjective attributes such as patient-reported outcomes as part of the case definition could result in different diagnostic performance over the study arms.

Further, and as demonstrated by the examples, it should be noted that diagnostic performance of study endpoints is always a combination of case definition as a set of attributes and diagnostic tests verifying these attributes. The structures of case definitions in the trials presented were fundamentally different: The case definition of the first trial led to a very specific endpoint definition resulting in an unbiased efficacy estimation. The case definition of the second trial maximized the endpoint sensitivity and clearly reduced the endpoint specificity, necessarily resulting in biased efficacy estimation with a possible loss of statistical significance. Therefore, case definitions in clinical trials should focus on endpoint specificity in order to avoid bias or the possible necessity of adjusting for endpoint sensitivity and specificity requiring the evaluation of diagnostic sensitivity and specificity, which is a complex procedure by itself. To accomplish this, the diagnostic systems applied have to be taken into account in order to not lose overall specificity as a result of applying an unspecific diagnostic approach. The consequence could be differing case definitions depending on their use either in clinical practice or in clinical trials.

## Figures and Tables

**Table 1:** HIV prevention efficacy estimates depending on specificity assumptions using Equations (7) and (9).

Specificity Assumption	Efficacy Estimation	0.95 Confidence Interval
1	0.385	0.090, 0.584
0.995	0.408	0.094, 0.613
0.985	0.461	0.101, 0.677
0.975	0.532	0.102, 0.756
0.965	0.627	0.067, 0.851
0.955	0.764	−0.246, 0.955

**Table 2:** *Clostridium difficile* relapse prevention efficacy estimates depending on specificity assumptions using Equations (7) and (9).

Specificity Assumption	Efficacy Estimation	0.95 Confidence Interval
1	0.729	0.201, 0.908
0.98	0.751	0.172, 0.925
0.96	0.774	0.126, 0.942
0.94	0.798	0.048, 0.957
0.927	0.815	−0.031, 0.967
0.92	0.824	−0.090, 0.972
0.90	0.852	−0.370, 0.984
